# Incidental detection of paranasal sinuses abnormalities on CT imaging of the head in Saudi adult population

**DOI:** 10.1371/journal.pone.0270764

**Published:** 2022-09-02

**Authors:** Ali Hassan A. Ali, Omar O. Serhan, Mohammed H. Karrar Alsharif, Abubaker Y. Elamin, Sameer Al-Ghamdi, Khaled K. Aldossari, Naif Alrudian, Mansour Alajmi, Bader A. Alhariqi, Mohammad Mokhatrish, Velmurugan Palanivel

**Affiliations:** 1 Anatomy Department, College of Medicine, Prince Sattam Bin Abdulaziz University, Al-Kharj, Saudi Arabia; 2 Anatomy Department, Faculty of Medicine, Al-Azhar University, Cairo, Egypt; 3 Radiology Department, King Khalid Hospital, Ministry of Health, Al-Kharj, Saudi Arabia; 4 Histology & Embryology Department, Faculty of Medicine, Ondukuz Mayis University, Samsun, Turkey; 5 Family & Community Medicine Department, College of Medicine, Prince Sattam Bin Abdulaziz University, Al-Kharj, Saudi Arabia; 6 Department of Family and Community Medicine, College of Medicine, Prince Sattam Bin Abdulaziz University, Al Kharj, Saudi Arabia; 7 Radiology Department, College of Medicine, Prince Sattam Bin Abdulaziz University, Al-Kharj, Saudi Arabia; 8 Surgery Department, College of Medicine, Prince Sattam Bin Abdulaziz University, Al-Kharj, Saudi Arabia; 9 Centre for Materials Engineering and Regenerative Medicine, Bharath Institute of Higher Education and Research (BIHER), Chennai, Tamil Nadu, India; Universiti Putra Malaysia, MALAYSIA

## Abstract

The paranasal sinuses are hollowed, air-filled cavities surrounding the nasal cavity. Many pathological processes affect the sinuses, but inflammatory conditions are the commonest, even in asymptomatic patients who undergo head imaging for other indications showing one or more abnormalities of the sinuses. Our research aims to determine the prevalence of incidental paranasal sinuses abnormalities seen among patients who undergo head CT scanning. In addition, it provides baseline information for further investigations required. The study was designed to evaluate all patients who underwent head CT scanning for any reason unrelated to paranasal sinuses abnormalities. 1849 cases were selected and retrospectively analyzed from the elective and emergency CT in the last nine months, from August 2020 to April 2021. In order to meet the inclusion criteria, indications for imaging must not be sinus-related. The study was conducted on 1849 cases who had undergone head CT scans for pathology, 1204 (65%) were male and 645 (35%) were female. Abnormalities of the sinuses were found in about 617 (33%) of all patients, with a higher rate in males (22.23%) than females (11.14%). In addition, these abnormalities were found in younger patients at a higher rate than in middle and old ages 19.74%, 7.19%, and 6.44%, respectively. Our findings revealed that the prevalence of paranasal sinuses abnormalities in asymptomatic Saudi patients was high (33%). Most of the affected sinuses were the maxillary. The male patients were more affected than females in all findings.

## Introduction

Paranasal sinuses are air-filled chambers that act as extensions of nasal cavities. They are named after the bones in which they are located: frontal, sphenoid, ethmoid & maxillary [1].

All are absent or rudimentary at birth. Start to develop in the first two years after birth. They enlarge, especially during the eruption of permanent teeth at puberty [2, 3].

Inflammatory disease is the most common among the pathologies involving the paranasal sinuses. Mild mucosal thickening, primarily within the maxillary and ethmoid sinuses, is common even in asymptomatic individuals. In contrast, acute sinusitis is characterized by the presence of air-fluid levels or foamy-appearing sinus secretions and is typically caused by a viral upper respiratory tract infection. On the other hand, in chronic sinusitis, changes include mucoperiosteal thickening and bony thickening of the sinuses’ wall [4].

Incidental findings are defined as findings that appear unrelated to the clinical indication for imaging purposes. The clinicians should understand the incidental findings and study head and neck anatomy to avoid misinterpretations [5].

Many studies showed that incidental paranasal sinus abnormalities are common findings on MRI images done for other problems. Among the commonly detected abnormalities, mucosal thickening involving the ethmoidal & maxillary sinuses is the highest. Up to 6mm thickness was seen in asymptomatic individuals. Hence, it is recommended that reporting such findings is unnecessary unless there are accompanying clinical symptoms [6–9].

Previous studies on various population subsets have reported the prevalence of sinus abnormalities to range from 16% to 60% [10, 11]. Another retrospective study carried out to determine the prevalence of abnormality in the paranasal sinuses in the British population having MRI scans for neurological signs & symptoms revealed that among 130 patients studied, 49.2% showed one or more abnormalities [8].

According to the American Academy of Allergy, the person suffering from sinusitis misses 4 to 6 workdays per year, and that directly affects the kingdom’s economy. Furthermore, regarding the social complications, sinusitis has a negative impact on the patient and family’s quality of life. This represents emotional distress, declining normal daily activities, and reduced attendance at school.

The information about sinusitis as a medical condition in the Kingdom of Saudi Arabia is considered few. And all the above-mentioned factors highlight the importance of this research and its value since setting a medical protocol for the diagnosis through computed tomography imaging will help in the treatment of sinusitis and, on the other hand, will contribute to an active role in reducing the aforementioned problems in the Saudi adult population. In our research, we will obtain statistical data about sinusitis among the Saudi population, identify the commonest paranasal sinuses involved in sinusitis, establish the causes of sinusitis among the Saudi population, and determine if the genetic and environmental factors play a role in influencing sinusitis.

Negligence of diagnosis and treatment of sinusitis may lead to impairment or partial loss of sense of smell and a great effect on the patient quality of life. This condition may lead to emotional distress, a decline in normal daily activities, and reduced attendance at school or work.

The objective of the study is to determine the prevalence of incidental paranasal sinuses abnormalities seen among patients who undergo head CT scanning. In addition, it provides baseline information for further investigations required.

## Methods

This Retrospective study was conducted in the department of radiology at King Khalid Hospital in Al-Kharj. The CT scan was performed at the department of radiology. All patients (males and females) who underwent head CT scanning for indications unrelated to paranasal sinuses abnormalities were included in this study.

Patients who have a clinical diagnosis of acute or chronic sinusitis and those with severe head and facial trauma were excluded. Young patients below 19 years and very old above 90 were also excluded from this study.

Ethical clearance was obtained and approved by the Institutional Review Board from Prince Sattam bin Abdul Aziz University (2020/03/17094). The confidentiality of the participants was secured. The total sample population was 1850 patients undergoing CT scans. The patients were selected and retrospectively analyzed from the elective and emergency CT in the last nine months, from August 2020 to April 2021. In order to meet the inclusion criteria, indications for imaging must not be sinus-related.

Computed tomography (CT) is the imaging method of choice for conditions that affect the paranasal sinuses. All scans were performed on the same 16-detector CT scanner, the Light speed 16 (GE Healthcare, Milwaukee, WI, USA). High-resolution axial images were obtained with the patient in the supine position. [12, 13].

For CT image evaluations, proprietary software from the manufacturer (Alisa MS DICOM Viewer/1. 7. 4 and Aliza Medical Imaging and DICOM Viewer 2.3.3) were used to analyze the images.

CT soft copy images of patients who will do head CT will be collected by the radiologists; then, we will analyze any presence of paranasal sinuses abnormalities by the investigator and the advising senior consultant radiologist. The features of paranasal sinuses, bony erosion, wall thickening, calcification, and tissue invasion, will be noted. Demographic data and radiological features regarding ethmoid and frontal sinus opacification, maxillary sinus bony erosion, size, wall thickening, calcification, and tissue invasion were assessed. The other abnormalities such as fluid levels, complete sinus opacification & presence of polyp or retention cyst were also collected. All scans were reviewed by two radiologists under standard soft-tissue and bone windows.

Data Entry & Analysis: The anatomical location & type of paranasal sinuses abnormalities identified were coded. Then the quantitative data was entered & analyzed using the latest version of Statistical Package Software IBM SPSS version 22 (SPSS, Inc. Chicago, IL). By doing so, the frequency of each type of abnormality was calculated. It was also analyzed if there was any correlation between the variables.

We will also use a student t-test to achieve comparison of the mean age of patients with incidental findings. Other statistical evidence of associations on categorical variables will be computed from Chi-square tests of contingency. A P-value of <0.05 will be considered significant.

## Results

**Fig 1:**
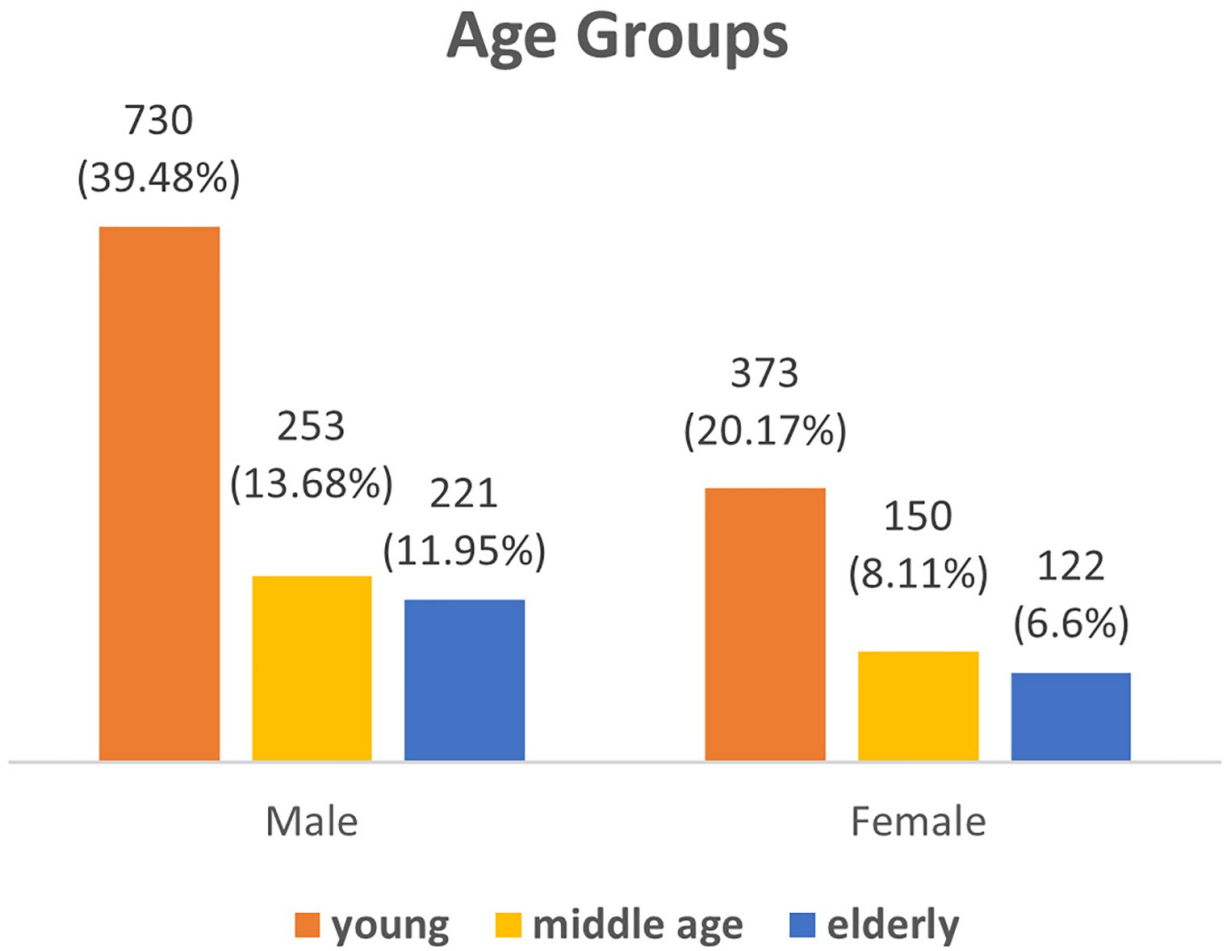
Distribution of the age groups.

**Fig 2:**
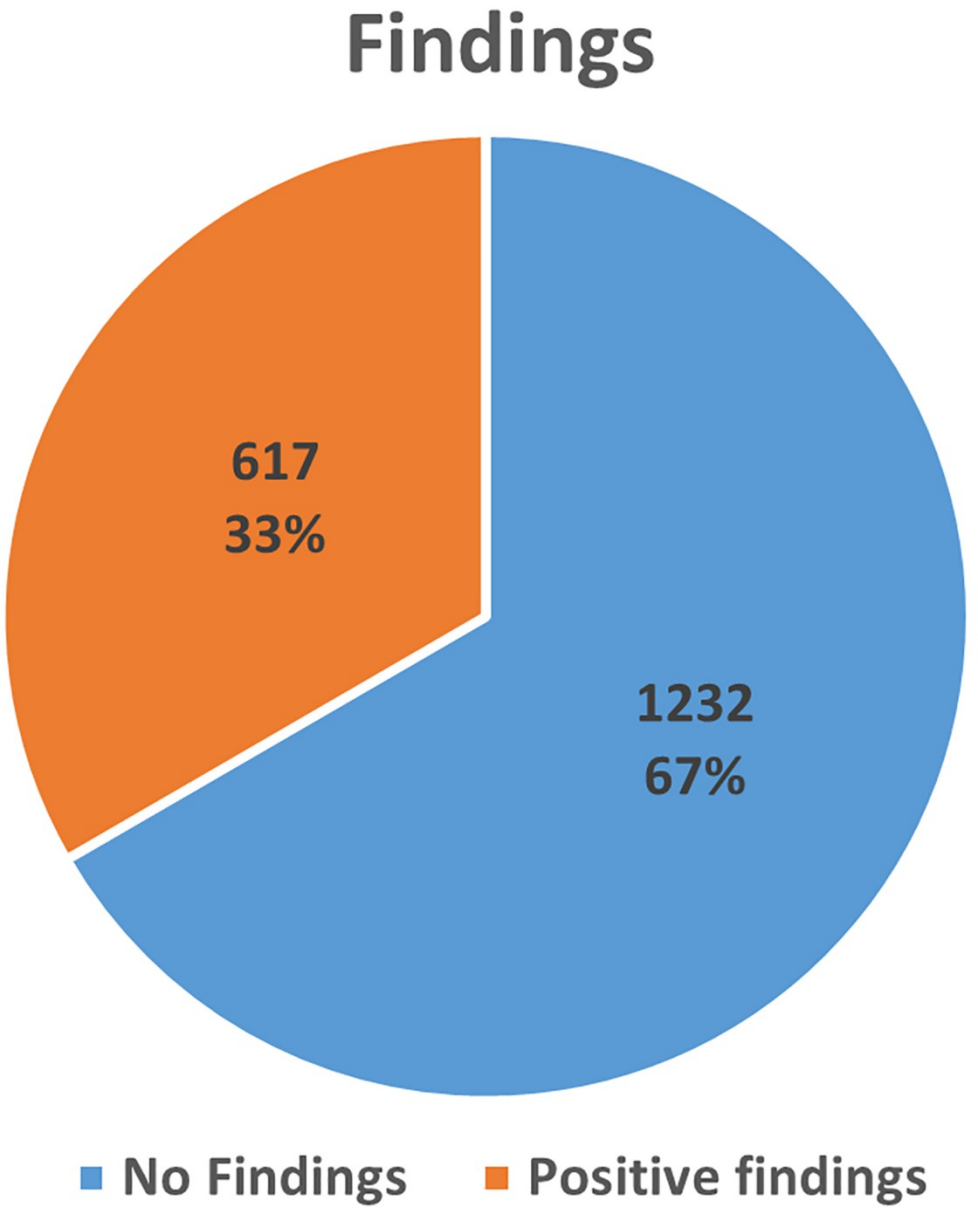
Prevalence of the paranasal sinuses among the patients. After assessing the CT scans, the data showed that the most frequently affected were maxillary sinuses, with a total (31.7%, n = 586) of all positive cases. unilateral maxillary antrum sinusitis was the most frequent pathologies (20.49%, n = 249) of all positive findings, maxillary polyps (Fig 3).

Analyzing CT scans of 1849 cases made by radiologists for pathology, 1204 (65%) were male and 645 (35%) were female. The age ranged from 17 to 90 years, with a mean age of 45.16 ± 18.96 SD. Abnormalities in the sinuses were found in about 617 (33%) of all patients with a higher rate in males (22.23%) than females (11.14%). To some extent, these abnormalities were found in younger patients at a higher rate than in middle and old age, 19.74%, 7.19% and 6.44% respectively (Figs 1 and 2).

**Fig 3:**
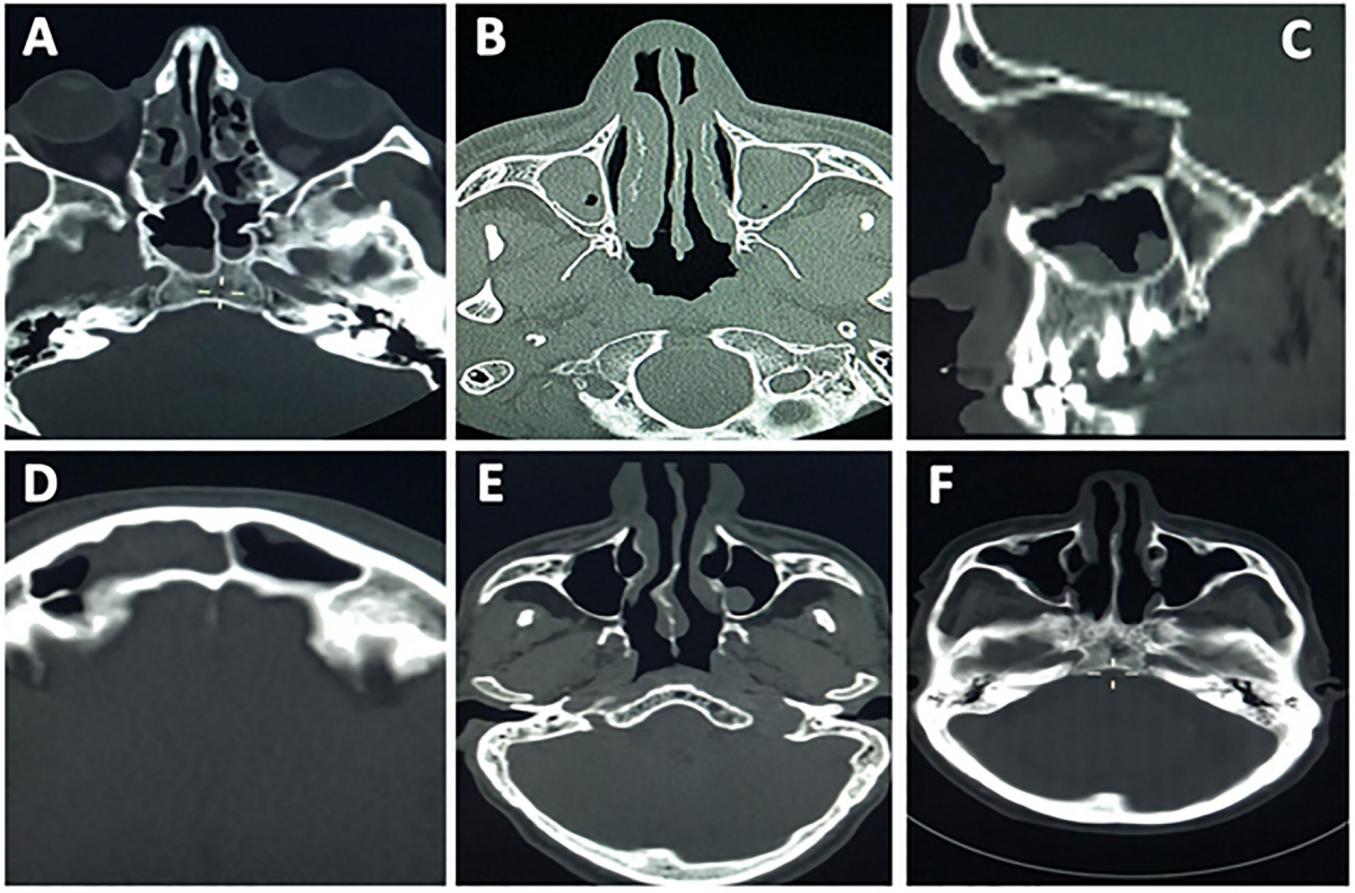
Patients brain CT scans show the incidental detection of paranasal sinuses abnormalities; (A) right maxillary antrum polyp, (B) bilateral maxillary polyp with retention cyst, (C) maxillary antrum polyp and right nasal air fluid level likely abscess, (D) right frontal sinusitis, (E) normal paranasal study, (F) right maxillary antrum mucosal thickening.

Bilateral maxillary antrum sinusitis and maxillary cyst were seen as positive but in lower percentage (11.89%, n = 144, 8.07%, n = 89, 7.82%, n = 95). Other sinuses were found to have a high percentage; partial ethmoiditis (16.3%, n = 198), sphenoidal sinusitis (13.9%, n = 195) frontal sinusitis (11.6%, n = 141) and nasal septum deviation (10.78%, n = 249) (Table 1).

**Table 1:** Shows the frequencies and percentage of the sociodemographic data as well as the pathological findings of the positive cases.

		Frequencies (%)
**Gender**	Male	411 (22.23%)
	Female	206 (11.14%)
**Age groups**	young 15–30	365 (19.74%)
	middle age 31–50	133 (7.19%)
	Elderly >51	119 (6.44%)
**Partial Ethmoiditis**		198 (10.71%)
**Unilateral Maxillary Antrum Sinusitis**		249 (13.47%)
**Bilateral Maxillary Antrum Sinusitis**		98 (5.3%)
**Frontal Sinusitis**		141 (7.63%)
**Sphenoidal Sinusitis**		159 (8.6%)
**Nasal Septum Deviation**		131 (7.08%)
**Maxillary cyst**		95 (5.14%)
**Maxillary polyps**		144 (7.79%)

The data shows that the male patients were more affected than females in all findings (Table 2) but only nasal septum findings showed a significant association between the males and females.

**Table 2:** Shows an association between gender and the types of paranasal sinusitis. It represents the frequency (%), and yes/no indicates the presence/absence of the abnormality. For the positive findings section, the patient was considered positive if he/she expressed one or more abnormalities.

		Male	Female	Chi-square Sig.
**Positive Findings**	No	793 (42.89%)	439 (23.74%)	.339
	Yes	411 (22.23%)	206 (11.14%)	
**Paranasal Abnormality**				
**Partial Ethmoiditis**	No	1079 (58.36%)	572 (30.94%)	.535
	Yes	125 (6.76%)	73 (3.95%)	
**Unilateral Maxillary Antrum Sinusitis**	No	1035 (55.98%)	565 (30.56%)	.327
	Yes	169 (9.14%)	80 (4.33%)	
**Bilateral Maxillary Antrum Sinusitis**	No	1143 (61.82%)	608 (32.88%)	.540
	Yes	61 (3.3%)	37 (2%)	
**Frontal Sinusitis**	No	1115 (60.3%)	593 (32.07%)	.605
	Yes	89 (4.81%)	52 (2.81%)	
**Sphenoidal Sinusitis**	No	1100 (59.49%)	590 (31.91%)	.935
	Yes	104 (5.62%)	55 (2.97%)	
**Nasal Septum Deviation**	No	1108 (59.92%)	610 (32.99%)	.042*
	Yes	96 (5.19%)	35 (1.89%)	
**Maxillary cyst**	No	1146 (61.98%)	608 (32.88%)	.394
	Yes	58 (3.14%)	37 (2%)	
**Maxillary polyps**	No	1105 (59.76%)	600 (32.45%)	.341
	Yes	99 (5.35%)	45 (2.43%)	

*. The Chi-square statistic is significant at the .05 level.

The data also revealed that the young patients were the most affected among age groups (365 cases); the highest abnormality was found in unilateral maxillary antrum sinusitis and partial ethmoiditis 146 (7.9%) and 130 (7.03%), respectively (Table 3). The data also showed that there is a significant association between the age groups and frontal sinusitis (P-value 0.000) and nasal septum deviation (P-value 0.000).

**Table 3:** Shows the association between the age group and the types of paranasal sinusitis. It represents the frequency (%), and yes/no indicates the presence/absence of the abnormality.

		young	middle age	elderly	Chi-square Sig.
**Partial Ethmoiditis**	No	363 (19.63%)	363 (19.63%)	315 (17.04%)	.141
	Yes	130 (7.03%)	40 (2.16%)	28 (1.51%)	
**Unilateral Maxillary Antrum Sinusitis**	No	350 (18.93%)	350 (18.93%)	293 (15.85%)	.800
	Yes	146 (7.9%)	53 (2.87%)	50 (2.7%)	
**Bilateral Maxillary Antrum Sinusitis**	No	389 (21.04%)	389 (21.04%)	327 (17.69%)	.100
	Yes	68 (3.68%)	14 (0.76%)	16 (0.87%)	
**Frontal Sinusitis**	No	372 (20.12%)	372 (20.12%)	300 (16.22%)	.000*
	Yes	67 (3.62%)	31 (1.68%)	43 (2.33%)	
**Sphenoidal Sinusitis**	No	373 (20.17%)	373 (20.17%)	318 (17.2%)	.301
	Yes	104 (5.62%)	30 (1.62%)	25 (1.35%)	
**Nasal Septum Deviation**	No	394 (21.31%)	394 (21.31%)	341 (18.44%)	.000*
	Yes	120 (6.49%)	9 (0.49%)	2 (0.11%)	
**Maxillary cyst**	No	380 (20.55%)	380 (20.55%)	332 (17.96%)	.198
	Yes	61 (3.3%)	23 (1.24%)	11 (0.59%)	
**Maxillary polyps**	No	381 (20.61%)	381 (20.61%)	311 (16.82%)	.111
	Yes	90 (4.87%)	22 (1.19%)	32 (1.73%)	

*. The Chi-square statistic is highly significant at the .005 level.

## Discussion

In reviewing the literature, morphological changes in paranasal sinus are common findings on head C.T and MRI 9. Our findings revealed that the prevalence of the paranasal sinuses is high at 33%. A comparison of the findings with those of other studies confirms that the percentage of asymptomatic patients who had sinus abnormality is high [14–17].

The data shows that the young patients are the most affected by the asymptomatic sinuses’ abnormalities, and the males, to a great extent, are more affected than the females.

Our data also shows that the maxillary sinuses were the most affected with pathologies than ethmoidal, sphenoidal and frontal sinuses. This finding broadly supports the work of other studies in this area [18, 19].

Of all maxillary sinuses’ abnormalities, unilateral maxillary antrum sinusitis was the highest; although the maxillary cysts are common in the maxillary sinuses, it appears to be low in our findings (7.82%). and more frequently were the maxillary polyps (11.85); these findings are consistent with the literature [19].

A previous study on the maxillary sinuses of 1442 Saudi patients by Ali revealed that about 495 (34.33%) of the cases had positive results, with the majority of them being males [20].

On the other hand, a previous thesis was performed at Addis Ababa University on the incidental detection of paranasal sinuses abnormalities. They showed that mucosal thickening was the highest, followed by retention cysts. The least frequent abnormality was sinus opacity [19, 21, 22].

In accordance with the present results, previous studies have demonstrated that There was no significant relationship between paranasal sinus abnormalities and sex and age 9. With an exception to nasal septum deviation, which showed a significant association between the males and females as well as age groups. and there was also a highly significant correlation between the age groups and frontal sinusitis. All other findings revealed no clinical association.

## Conclusion

Our findings revealed that the prevalence of paranasal sinuses abnormalities in asymptomatic Saudi patients was high at 33%. Most of the affected sinuses were the maxillary. The male patients were more affected than females in all findings. On the other hand, clinicians should be aware of the anatomical differences in incidental findings to reduce further unnecessary diagnostic evaluations and allow the selection of more appropriate treatment plans. It is also necessary for comprehensive health care for their patients.

For that reason, Careful observation is necessary during CT scans of the head. This led to early identification, management and follow up of sinus abnormalities.
